# Shenshuaikang enema restores the intestinal barrier and microbiota-gut-kidney axis balance to alleviate chronic kidney disease via NF-κB pathway

**DOI:** 10.3389/fphar.2024.1453668

**Published:** 2025-01-21

**Authors:** Yan Ye, Xiaopeng Huang, Xueying Li, Fei Gao, Wenzhen Zhong, Anqi Tang, Liangbin Zhao, Dengpiao Xie, Naijing Ye

**Affiliations:** ^1^ TCM Regulating Metabolic Diseases Key Laboratory of Sichuan Province, Hospital of Chengdu University of Traditional Chinese Medicine, Chengdu, China; ^2^ Hospital of Chengdu University of Traditional Chinese Medicine, Chengdu, China; ^3^ Department of Ministry of Science, Hospital of Chengdu University of Traditional Chinese Medicine, Chengdu, China; ^4^ School of Clinical Medicine, Chengdu University of Traditional Chinese Medicine, Chengdu, China; ^5^ State Key Laboratory of Southwestern Chinese Medicine Resources, Chengdu University of Traditional Chinese Medicine, Chengdu, China

**Keywords:** chronic kidney disease (CKD), shenshuaikang enema (SSKE), microbiota-gutkidney axis, NF-κB pathway, intestinal barrier

## Abstract

**Introduction:**

Chronic kidney disease (CKD) is a chronic progressive disease characterized by abnormalities in kidney structure or function caused by variousfactors. It has become a significant public health problem, posing a threat to human health worldwide. Shenshuaikang enema (SSKE) has demonstrated notable efficacy and safety in treating CKD, although its mechanism of action remains unclear.

**Methods:**

The CKD rat model was induced using 2.5% adenine, and the effect of SSKE was evaluated by detecting uremic toxins, inflammatory cytokines, and renal function. The structure of the intestine and kidney was observed using electron microscopy. Pathological changes in the intestine and kidney were detected by H&E staining. The expression of Occludin, Claudin-1, and ZO-1 in the intestine was detected by immunohistochemistry. The degree of renal fibrosis was observed using Masson and PAS staining. The expression of NF-κB and MyD88 protein in the intestine, and the expression of F4/80, TLR4, NF-κB and MyD88 in the kidney were detected by immunofluorescence staining. NF-κB-RE-Luc transgenic mice were used to construct a CKD mouse model, and changes in fluorescence intensity in mice and isolated kidney tissues were detected within 1–6 days using a small animal live imager. Finally, 16S rRNA amplicon sequencing was used to monitor changes in intestinal flora in CKD patients before and after SSKE treatment.

**Results:**

We found that SSKE improves renal function, attenuates renal fibrosis, reduces inflammatory factor levels, and decreases damage to intestinal and renal structures in adenine-induced CKD rats. Additionally, our results suggest that SSKE regulates NF-κB pathways, increases the expression of tight junction proteins, improves intestinal permeability, promotes the growth of beneficial bacteria, inhibits the proliferation of harmful bacteria, and reduces metabolic disorders. Ultimately, these effects contribute to the efficacy of SSKE in treating CKD.

**Conclusion:**

These results indicate that SSKE restores intestinal barrier function by regulating the microbiota-gut-kidney axis, thereby treating CKD.

## 1 Introduction

Chronic kidney disease (CKD) is a chronic progressive disease characterized by abnormalities in the structure or function of the kidneys, mainly evidenced by a decrease in the estimated glomerular filtration rate (eGFR) (Chen T. K. et al., 2023; Kanbay et al., 2024). Currently, CKD has emerged as a significant threat to human health, following cardiovascular and cerebrovascular diseases, cancers, and diabetes (Liu et al., 2022). CKD represents a global public health problem because of its high incidence, low awareness rate, poor prognosis, and high medical expenses. It severely impacts the quality of life of patients and their families and increases the burden on both families and society (Banerjee et al., 2021; Jadoul et al., 2024; Luyckx et al., 2020).

In 2011, Meijers introduced the concept of the “gut-kidney axis,” which innovatively linked the interaction between gut microbiota and the kidneys (Meijers and Evenepoel, 2011). In recent years, research on the gut-kidney interaction in CKD has been on the rise (Ai et al., 2023; Pan et al., 2023; Shen et al., 2023). Studies have shown that CKD leads to disruptions in intestinal flora and metabolic disorders (Holle et al., 2022; Sarakpi et al., 2023; Sohn et al., 2024). Concurrently, disorders in the intestinal flora and the accumulation of uremic toxins can damage the intestinal barrier function in CKD patients (Beker et al., 2022; Wang et al., 2024; Ye et al., 2023). Moreover, the impaired intestinal barrier further exacerbates the translocation of bacteria and toxins, worsening systemic inflammation in patients (Ji et al., 2020; Wu et al., 2022). Therefore, targeting and regulating gut microbiota holds significant potential for the treatment of CKD.

Traditional Chinese medicine (TCM) is widely used as a complementary therapy for CKD, addressing interactions between the intestines and kidneys (Zhao et al., 2020). According to the classical theory of TCM, CKD arises from the pathogenesis of “deficiency, stasis, dampness, and toxicity” (Wang et al., 2022). Based on this theory, we propose a Chinese herbal formula called Shenshuaikang Enema (SSKE), composed of Astragalus membranaceus, Rheum palmatum, Salvia miltiorrhiza, and Carthamus tinctorius, to “benefit qi, activate blood circulation, and dispel dampness and detoxification” (Lu et al., 2020). In our previous study, SSKE was shown to reduce urinary albumin excretion and inflammatory markers in CKD mice after 4 weeks of administration (Lu et al., 2020). The effective constituents of rhubarb (emodin, chrysophanol, etc.) have also been found to improve intestinal barrier function, regulate intestinal microbial disorders, and affect the expression of key proteins in the intestinal NF-κB and Keap1/Nrf2 signalling pathways to alleviate renal dysfunction and tubulointerstitial fibrosis (Gu et al., 2022; Xu et al., 2022). Based on these findings, we propose the hypothesis that SSKE is a novel approach for the treatment of CKD, with its molecular mechanism related to its ability to combat oxidative stress.

In this study, we investigated the protective effects of SSKE on CKD-related symptoms using various pharmacological approaches and CKD rat models. The results showed that SSKE treatment promoted renal protection and intestinal barrier repair by inhibiting the NF-κB pathway. Additionally, SSKE improved the intestinal microbial community in CKD patients. This study aims to provide further evidence supporting the effectiveness of SSKE in managing CKD.

## 2 Materials and methods

### 2.1 Materials and chemicals

According to the Chinese Pharmacopoeia (2020 Edition), the four Chinese herbal pieces of SSKE were identified by Dr. Yan Ye. Adenine (Sigma-Aldrich LLC.), enzyme-linked immunoassay (ELISA) kits (MultiScience (Lianke) Biotech Co., Ltd.), methanol (Sigma-Aldrich LLC.), phosphoric acid (Chengdu Chron Chemical Co., Ltd.) were used. Anti-Occludin rabbit antibody, anti-Claudin-1 rabbit antibody, anti-ZO-1occludin rabbit antibody (Protein-tech Group, Inc.), 4′,6-diamidino-2-phenylindole (DAPI, Sigma-Aldrich Co. LLC.) were used. A polyclonal rabbit antibody against Toll-like receptor 4 (TLR4), anti-Myeloid differentiation primary response 88 (MyD88), anti- Nuclear Factor-kappa B (NF-κB) (Protein-tech Group, Inc.) was used.

### 2.2 HPLC analysis of the alcohol extract of SSKE

#### 2.2.1 SSKN preparation

The herbal decoction SSKE combines four medicinal herbs including *Astragalus membranaceus*, *Rheum palmatum*, *Salvia miltiorrhiza* and *Carthamus tinctorius* (detailed information provided in Supplementary Table S1) were obtained from the Hospital of Chengdu University of Traditional Chinese Medicine. The ingredients were soaked in distilled water (×8) for 1 h and decocted to 5 mL. The resulting solution was then filtered through two layers of gauze.

#### 2.2.2 Chromatographic conditions

Agilent TC-C18 (4.6 × 250 mm, 5 μm) was performed for chromato-graphic separation. The distilled water containing 0.1% phosphoric acid was used as solvent A, and Methanol was used as solvent B. Detail in-formation of HPLC operating parameters are outlined in Supplementary Table S2.

### 2.3 Animal model construction and administration

Male Sprague-Dawley rats (6–8 weeks, 220–240 g) were obtained from SPF Biotechnology Co., Ltd. (Beijing, China) and kept in a standard condition (22–25°C, 50%–60% humidity, 12 h/12 h light/shadow cycle). All animal procedures were conducted in accordance with the strict Guidelines for the Care and Use of Laboratory Animals of the Ministry of Science and Technology of China.

The adenine-induced CKD rat model was performed for treatment evaluation (Hayeeawaema et al., 2023; Zhao et al., 2024). The rats were randomly separated into five groups: 1) control group (saline); 2) model group (200 mg/kg/d adenine); 3) SSKE-L group (2.5 mL/kg/d); 4) SSKE-M group (5 mL/kg/d); 5) SSKE-H group (10 mL/kg/d). The model and SSKE groups involved initial oral administration of a 2.5% adenine (Sigma-Aldrich LLC.) suspension at a dosage of 200 mg/kg for the first 4 weeks, followed by the same dose administered orally every other day for the subsequent 3 weeks. The control group received equivalent volumes of saline. During the modelling period, SSKE was administered by enema, and saline enema was given to the control and model groups.

### 2.4 Biochemical tests

Peripheral blood was collected from mice, and enzyme-linked immunoassay (ELISA) kits were used to determine the levels of the gut-derived endotoxin indole including sulphate (IS) and lipopolysaccharide (LPS), as well as pro-inflammatory cytokines (TNF-α, IL-6) in each group of rats. Blood urea nitrogen (BUN) was detected by automatic biochemical analyzer (Cobas C 311); urine protein concentration was detected by bicinchoninic acid colorimetric method using automatic analyzer (BS-240VET).

### 2.5 Transmission electron microscope

To analyze ultrastructural changes in the gut and glomerulus, fresh intestinal and renal cortical tissue (<1 mm^3^) was fixed in electron microscope fixative for 2–4 h at 4°C, then, rinsed three times with 0.1 M phosphate buffer PB (pH 7.4). 1% osmium acid-0.1 M phosphate buffer (pH 7.4) was fixed for 2 h at room temperature. The samples were dehydrated, osmotic embedding, sectioned and stained, and then viewed under a transmission electron microscope (TEM) (HT7700, HITACHI) and images were collected for analysis.

### 2.6 Histological analysis

To examine the intestinal and renal pathological changes of SSKE on CKD rats, we used hematoxylin and eosin (H&E) staining followed by observation using an optical microscope (CX33, Olympus, Tokyo, Japan). Meanwhile, for observing the degree of improvement of SSKE on renal fibrosis in CKD model rats, we used Masson’s Trichrome Staining (Masson) staining, periodic acid-Schiff (PAS) staining in accordance with the standard protocols.

### 2.7 Immunohistochemical staining

The paraffin sections of ileum and colon tissues were washed with PBS and fixed with 4% paraformaldehyde. Immunohistochemical staining was subjected to evaluate the expression of Occludin, Claudin-1, ZO-1 and DAPI (nuclear staining). After sealing, images were observed and captured with a fluorescence microscope (Nikon Eclipse C1, Japan).

### 2.8 Immunofluorescence staining

To confirm the role of SSKE in modulating the gut-kidney axis to ameliorate CKD through the NF-κB signaling pathway, we used immunofluorescence staining for correlative validation. Paraffin sections of ileum, colon and kidney were dewaxed, hydrated and antigenically repaired. After blocking with 10% normal goat serum, the sections were incubated in primary antibody at 4°C overnight. Cell nuclei were stained with DAPI. Then washed with PBS, added with autofluorescence quencher dropwise, and sealed with anti-fluorescence quenching sealer. After sealing, the slices were placed under a fluorescence microscope (Nikon Eclipse C1, Japan) for observation and acquisition of the corresponding images.

### 2.9 *In Vivo* biodistribution evaluation

In order to dynamically monitor the NF-κB fluorescence changes in the kidney tissues of animals *in vivo* after intervention with SSKE in a mouse CKD model. Fluorescently labelled NF-κB transgenic mice (NF-κB-RE-Luc) were used for observation after isoflurane gas anaesthesia in conjunction with a Caliper Life Sciences LIVIS Lumina Series (PerkinElmer, Waltham, MA). The CKD mouse model was induced by 2.5% adenine, and the mice were randomly separated into three groups, as follows: orally administered control, model, and SSKE. After rectal administration for 1, 2, 3, 4, 5, and 6 days, the changes in fluorescence intensity in mice were observed. The mice were sacrificed at the final endpoint after administration, and the kidney was obtained from the mice to evaluate the distribution in the damaged kidney.

### 2.10 16S rRNA analysis of fecal microflora

To investigate the role and significance of fecal microflora in the pathogenesis of CKD and the differences before and after treatment with SSKE. We selected 10 patients who were admitted to the Hospital of Chengdu University of Traditional Chinese Medicine with a diagnosis of CKD. The research followed guidelines of the Declaration of Helsinki and Tokyo for humans, and was approved by the institutional human experimentation committee, and that informed consent was obtained. Their fecal samples before and after treatment by SSKE were collected, and their microbial composition was analyzed by 16S rRNA sequencing. PCR amplification was performed, purified amplicons were pooled, and paired-end sequenced. Then, a library was established and the sequence was compared to the bacterial gene bank to analyze the species abundance and composition of the bacterial community and observe the changes between the fecal microflora before and after treatment.

### 2.11 Statistical analysis

Data were presented as mean ± SD. Comparisons between two groups were made using independent samples t-test, and comparisons between three or more groups were performed using one-way ANOVA followed by the Tukey test in GraphPad Prism 8.0. It was deemed statistically significant when *p* < 0.05.

## 3 Results

### 3.1 Representative HPLC chromatograms of SSKE extract

As shown in Supplementary Figure S1, five main compounds were identified in the HPLC (246 nm) profile of the SSKE extract based on the retention time of the authentic standards. These representative components include aloe-emodin, rhein, emodin, chrysophanol, and emodin methyl ether.

### 3.2 SSKE improves endotoxin, inflammation levels and renal function in CKD rats

**FIGURE 1:**
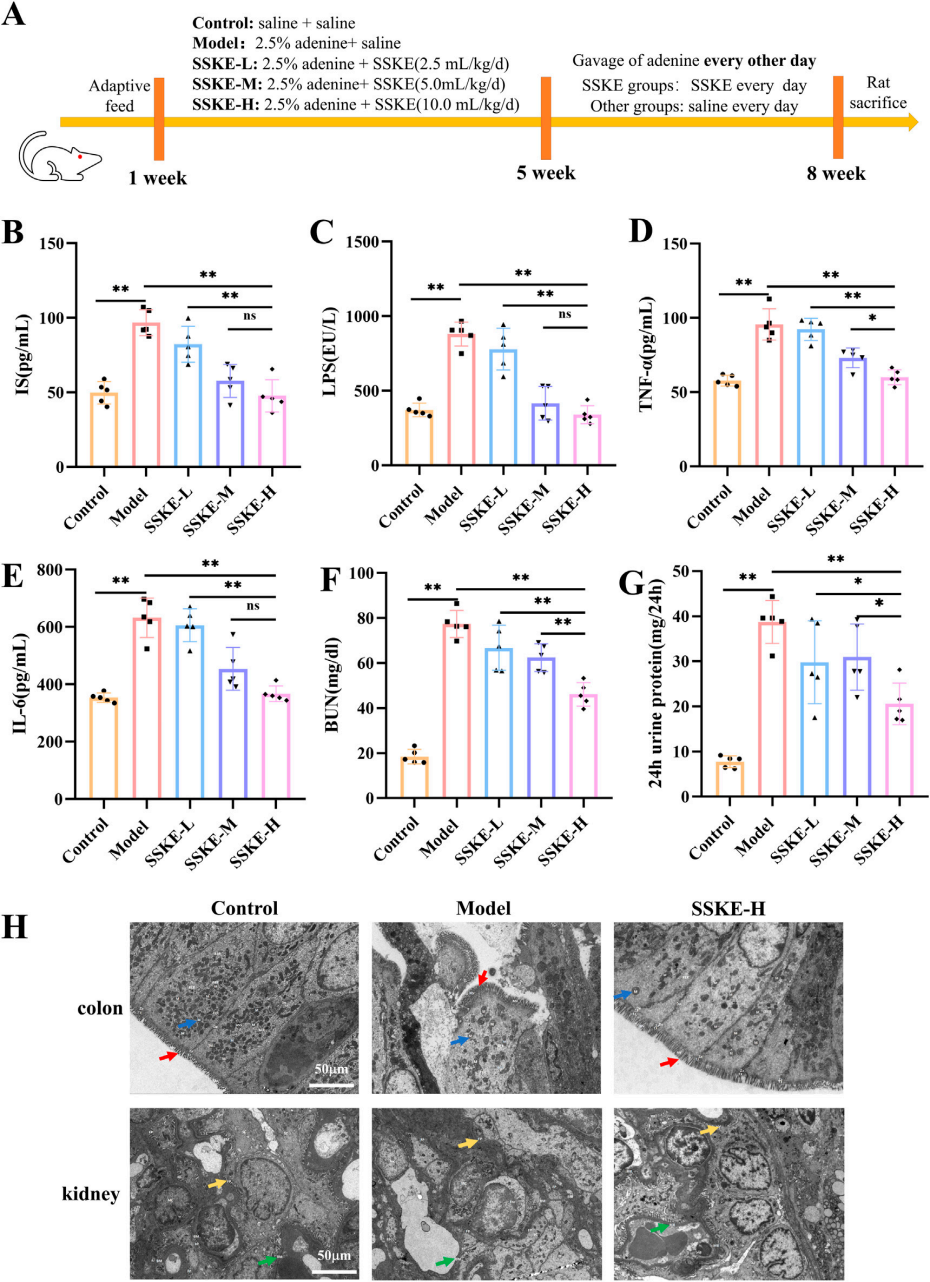
SSKE improves endotoxin, inflammation levels, renal function, and intestinal, renal ultrastructural damage in CKD rats. **(A)** Animal experimental protocol of this study. **(B–G)** The levels of IS, LPS, TNF-α, IL-6, BUN, 24 h urine protein in different groups. (n = 5). **(H)** Representative colon and kidney TEM of different groups. (n = 3). All data are presented as means ± SD. *p < 0.05; **p < 0.01.

In our study, the adenine-induced CKD rat model, a widely used model, was employed to explore the role of SSKE *in vivo*. The flowchart of the animal experiments is shown in Figure 1A. Intestinal endotoxin levels were detected by ELISA, and the results showed that (Figures 1B, C) compared with the model group, the SSKE-H group significantly reduced the levels of IS and LPS (*p* < 0.05), approaching the levels of the control group. The levels of inflammatory factors are shown in Figures 1D, E. Compared with the model group, SSKE-H significantly reduced the levels of TNF-α and IL-6 (*p* < 0.05). Further analysis of renal injury indicators in serum showed that (Figures 1F, G) compared with the model group, BUN and 24-h urine protein content decreased significantly after SSKE treatment, with SSKE-H showing the most prominent improvement effect (*p* < 0.05). These results indicate that SSKE can improve intestinal endotoxin, proinflammatory cytokines, and renal function in CKD rats, with SSKE-H having the best improvement effect. Therefore, some subsequent experiments only selected the SSKE-H treatment group for further research.

### 3.3 SSKE attenuates intestinal and renal ultrastructural damage in CKD rats

The gut-kidney axis has been identified as an important pathway for CKD treatment (Hsu et al., 2021). In this study, we explored the role of SSKE in treating CKD by examining both the intestine and kidney. TEM results (Figure 1H) showed that the model group exhibited severe damage to the intestinal barrier, including the loss of microvilli, incomplete cellular structures such as cytoplasmic membranes, mitochondria, and nuclei, and the loss of interstitial junctions, compared with the control group. In the SSKE-H group, the intestinal barrier was repaired, with microvilli, intracellular structures, and intercellular structures showing no significant damage. Additionally, in the model group, the glomerular basement membrane, podocytes, endothelial cells, and mesangial cells were damaged compared with the control group. These structures showed improvement in the SSKE-H group compared with the model group.

### 3.4 SSKE alleviated the pathological damage of intestinal and renal tissues in CKD rats

**FIGURE 2:**
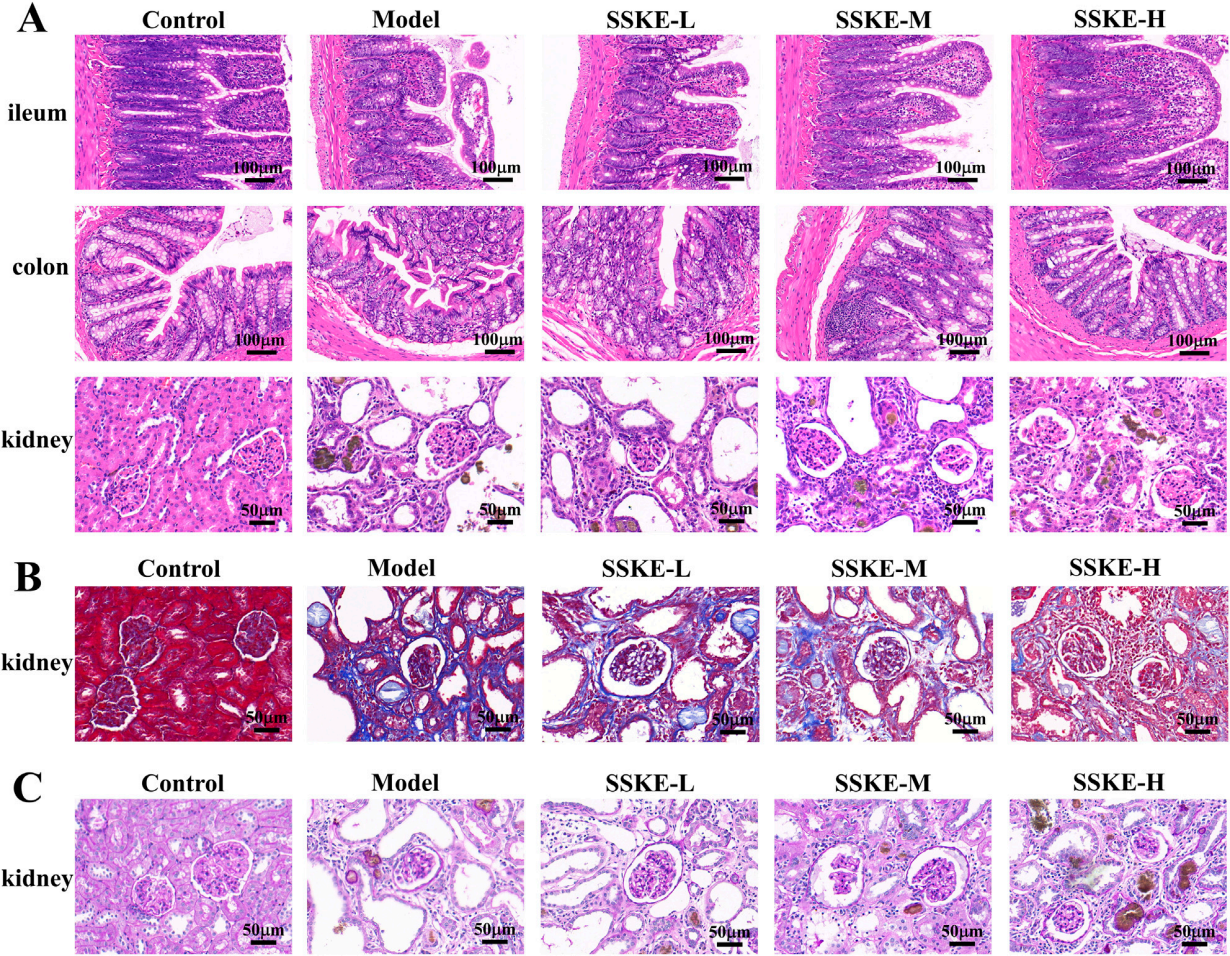
SSKE alleviated the pathological damage of intestinal and renal tissues in CKD rats. **(A)** H&E staining of ileum, colon and kidney in different groups. (n = 3). **(B, C)** Masson and PAS staining of kidney in different groups. (n = 3).

When CKD occurs, both intestinal and kidney tissues undergo varying degrees of damage (Sutthasupha et al., 2022). In this study, H&E staining results (Figure 2A) showed that, compared with the control group, the model group had sparse and shortened intestinal villi, inactive glandular proliferation, and significant mucosal edema. There was severe infiltration of inflammatory cells, disorganized and missing epithelial cells, twisted and fuzzy mucosal crypts, and sparse tissue structure. SSKE treatment reduced intestinal mucosal edema and inflammatory cell infiltration, restored intestinal epithelial cell arrangement, and improved mucosal crypt structure, indicating that SSKE can improve intestinal pathological structure. Additionally, in the model group, adenine metabolite crystals were deposited in the kidney, accompanied by extensive inflammatory cell infiltration, disordered glomerular structure, renal interstitial fibrosis, cystic dilatation of renal tubules, numerous lumen obstructions, and significant mesangial proliferation, consistent with the pathological changes observed in adenine model rats. SSKE treatment significantly alleviated renal pathological changes such as tubular lumen dilatation and inflammatory cell infiltration. Masson and PAS staining results (Figures 2B,C) showed that renal fibrosis was significantly increased in the model group compared with the control group. However, SSKE treatment reduced renal fibrosis compared with the model group. These analyses illustrate the ability of SSKE to reduce damage in renal tissue.

### 3.5 SSKE repairs intestinal mucosa by upregulating tight junction (TJs) proteins

**FIGURE 3:**
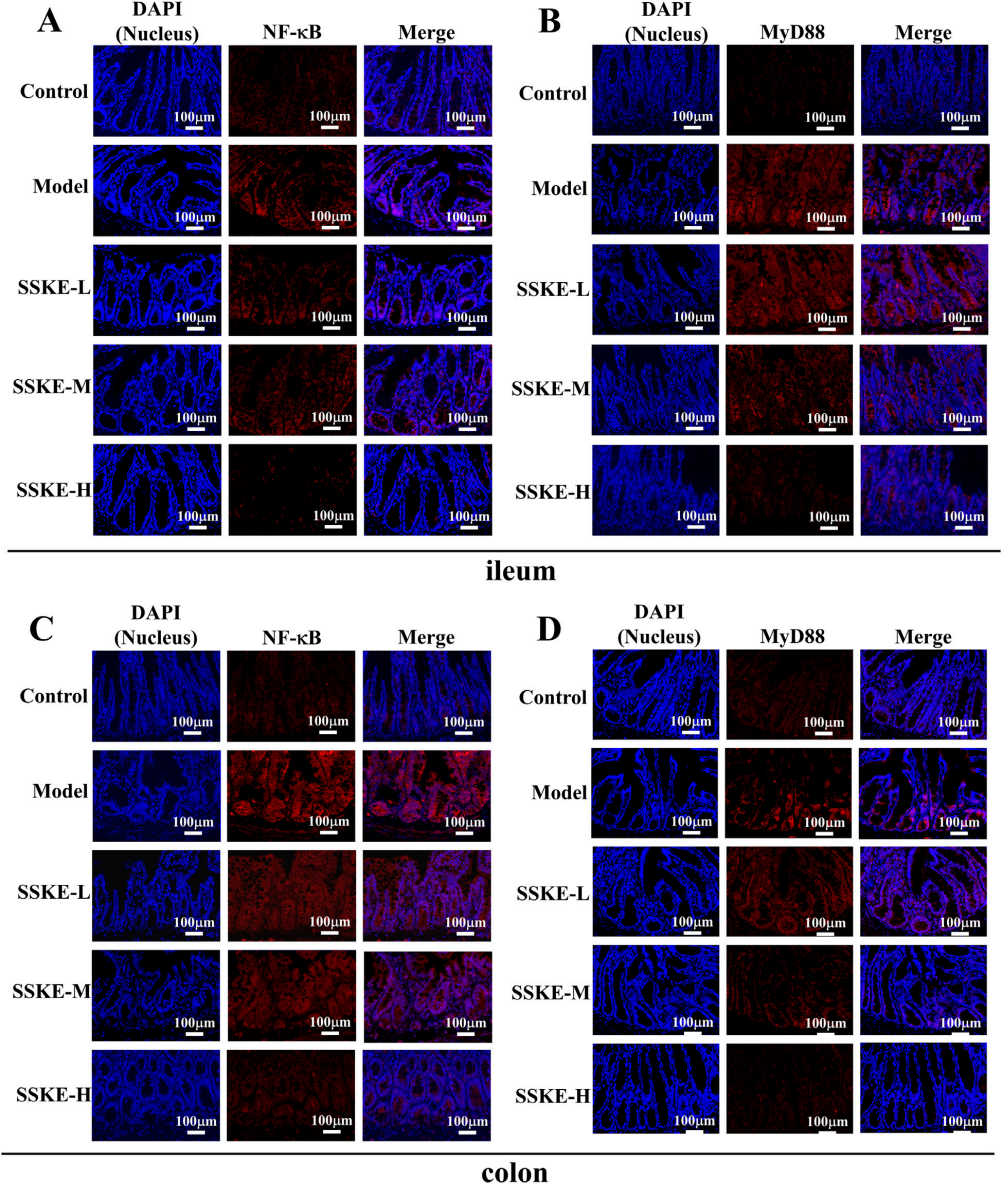
SSKE repairs intestinal mucosa by upregulating tight junction (TJs) proteins. **(A)** IHC of ileum in different groups. **(B)** IHC staining of colon in different groups. (n = 3).

Modern pharmacological studies have shown that intestinal epithelial barrier dysfunction plays a crucial role in the occurrence and progression of CKD (Georgia Andriana et al., 2023; Pyatchenkov et al., 2022). TJs are an essential component of the intestinal epithelial barrier, regulating its selective permeability and preventing harmful bacteria and toxins from entering the mucosa, which can lead to intestinal and systemic inflammation. Occludin, Claudin-1, and ZO-1 are key proteins that reflect intestinal barrier function. To verify the potential therapeutic effect of SSKE on CKD by improving intestinal epithelial barrier injury, we examined the expression levels of Occludin, Claudin-1, and ZO-1 using immunohistochemistry (IHC). The results (Figures 3A, B) showed that, compared with the control group, the expression of Occludin, Claudin-1, and ZO-1 decreased in the ileum and colon tissues of the model group, indicating severe damage to the intestinal barrier. However, the SSKE-H group exhibited higher expression levels of these three proteins in the ileum and colon compared to other treatment groups, suggesting a stronger protective effect on the TJs structure.

### 3.6 SSKE inhibits inflammatory response by inhibiting NF-κB signaling pathway

The severity of CKD is directly related to the amount of pro-inflammatory cytokines in the blood (Chen M. et al., 2023). Previous studies have confirmed that SSKE effectively reduces the production of pro-inflammatory cytokines. The activation of the NF-κB signaling pathway is crucial in the pathogenesis of CKD, promoting inflammation and regulating apoptosis and vascular remodeling. Our preliminary study suggests that SSKE’s therapeutic effect on adenine-induced CKD rats is associated with the inhibition of the NF-κB signaling pathway.

**FIGURE 4:**
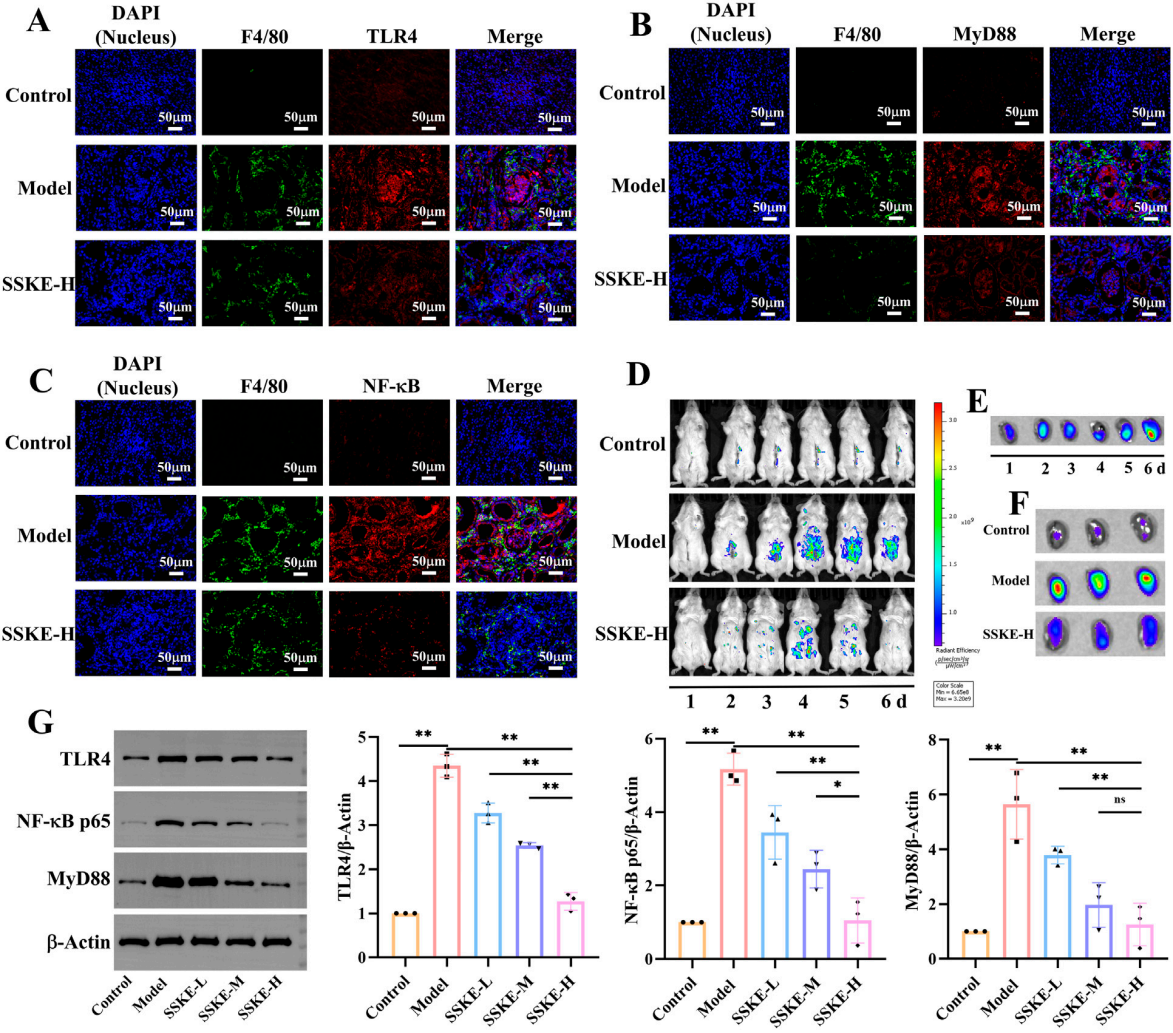
SSKE inhibits intestinal inflammatory response by inhibiting NF-κB signaling pathway. **(A, B)** IF of ileum in different groups. **(C, D)** IF of colon in different groups. (n = 3).

To further investigate the mechanism of SSKE in treating CKD via the gut-kidney axis, we evaluated the expression levels of proteins related to the NF-κB signaling pathway using immunofluorescence (IF). As shown in Figures 4A–D, compared with the control group, the fluorescence intensity of MyD88 and NF-κB increased in the ileum and colon tissues of the model group. The SSKE-H group showed significantly lower fluorescence intensity of MyD88 and NF-κB compared to the SSKE-L and SSKE-M groups.

**FIGURE 5:**
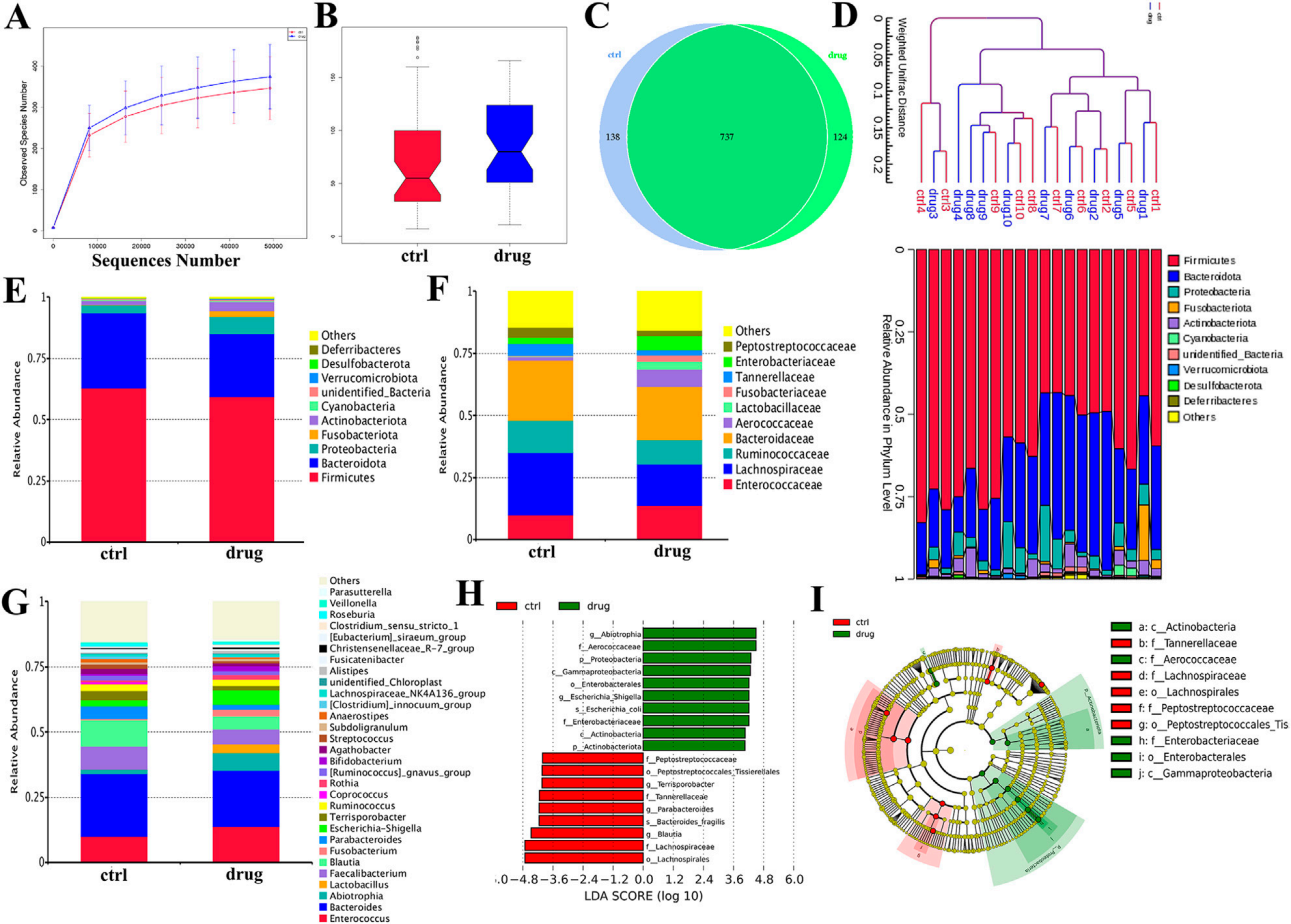
SSKE protects the kidney by inhibiting the NF-κB signalling pathway. **(A–C)** IF of kidney in different groups. **(D)** Fluorescence images of CKD mice at the different time points after SSKE administration. **(E)** The kidney fluorescence images of SSKE-H group at the different time point. **(F)** The kidney fluorescence images at the final time point.

Furthermore, we observed the expression levels of NF-κB signaling pathway-related proteins in kidney tissues and macrophage infiltration. As shown in Figures 5A–C, the model group exhibited increased fluorescence intensities of TLR4, MyD88, and NF-κB, as well as significant macrophage infiltration, compared with the control group. After SSKE-H treatment, the fluorescence intensity of TLR4, MyD88, and NF-κB decreased, and macrophage infiltration was reduced. This indicates that SSKE has potent anti-CKD effects by regulating the NF-κB signaling pathway.

To further examine the role of the NF-κB axis in CKD development and how SSKE can improve CKD by regulating the NF-κB axis, we used a small-animal *in vivo* imaging device to monitor the fluorescence intensity of NF-κB-RE-Luc BALB/c mice *in vivo* and *in vitro* kidney tissue in real time. As shown in Figures 5D–F, compared with the control group, the model group exhibited significantly enhanced fluorescence intensity of NF-κB *in vivo*, with a diffuse distribution in the intestinal area and an accumulation of NF-κB fluorescence intensity in isolated kidneys over time. In contrast, the SSKE treatment group showed weakened fluorescence intensity of NF-κB *in vivo* and in isolated kidneys, indicating that SSKE inhibits the expression of NF-κB.

### 3.7 SSKE restores intestinal flora homeostasis by regulating flora

The gut microbiota plays a crucial role in the pathogenesis of CKD, with microbial imbalances being a key factor in the disease’s onset and progression (Hsu and Tain, 2022; Mertowska et al., 2021). In this study, we used 16S rRNA gene sequencing technology to detect changes in the species composition and relative abundance of gut microbiota in CKD patients before and after SSKE treatment, evaluating SSKE’s regulatory effect on the gut microbiota.

**FIGURE 6:**
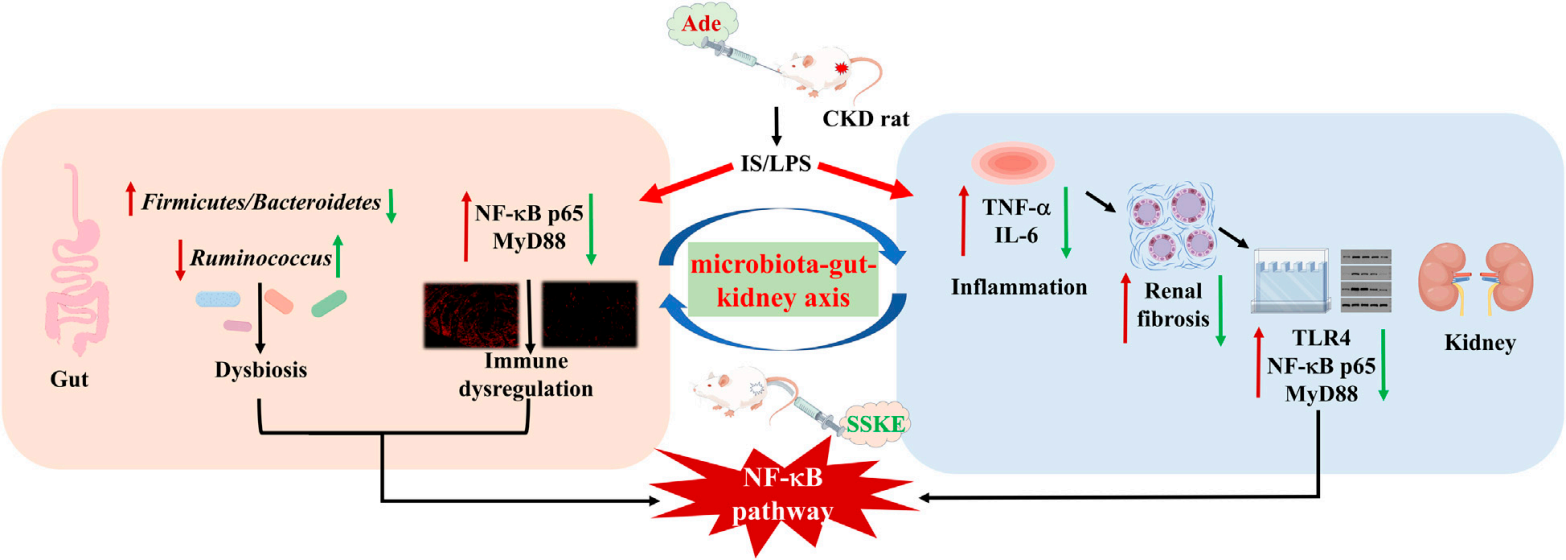
Evaluation of SSKE restoring the structure of gut microbiota in CKD patients. **(A)** α-diversity dilution curve. **(B)** β-diversity dilution curve. **(C)** Venn diagram. **(D)** Phylogenetic tree of abundance distribution. **(E)** The relative abundance of the top 10 species in each group at phylum among groups. **(F)** The relative abundance of the top 10 species in each group at family level. **(G)** The relative abundance of the top 20 species in each group at genus level. **(H, I)** Different gut microbiota composition profiles among groups and the Cladogram generated from the LEfSe analysis indicate the phylogenetic distribution from phylum to the genus of the microbiota. Histogram of LDA scores to identify differentially abundant bacterial genera (LDA score ≥3.0, Kruskal–Wallis = 0.05, Wilcoxon = 0.05).

First, we analyzed the diversity of bacterial communities in each group. The α-diversity dilution curve (Figure 6A) indicated that the gut flora in both groups had good diversity. The diversity analysis (Figure 6B) showed a biological difference between the CKD patient group (ctrl) and the treatment group (drug) (*p* < 0.5). To further investigate the microbial composition between the two groups, we created a Venn diagram (Figure 6C) to identify overlapping and unique taxa in each group. The results revealed 737 overlapping OTUs in both groups, with 138 unique OTUs in the ctrl group and 124 unique OTUs in the drug group. The phylogenetic tree (Figure 6D) provided further analysis of the species composition and community structure between the two groups, showing differences in microbial composition and structure. These findings indicate that SSKE can regulate the gut microbial levels of CKD patients.

Further, we used histograms to analyze the differences in species and relative abundance of gut microbiota in different groups at the phylum, family, and genus levels. At the phylum level (Figure 6E), the dominant bacteria mainly included *Firmicutes* and *Lactobacillus*. After SSKE treatment, the relative abundance of *Lactobacillus* was downregulated, consistent with other research findings. At the family level (Figure 6F), the abundance of Ruminococcaceae and *Lachnospiraceae* in the treatment group was lower than in CKD patients. The species composition at the genus level (Figure 6G) showed that SSKE treatment increased the relative abundance of *Lactobacillus*. LEfSe analysis (Figures 6H, I) identified a total of 19 differential bacterial genera between the two groups, with 9 genera significantly regulated by SSKE, including *Tannerellaceae*, *Lachnospiraceae*, *Lachnospirales*, *Peptostreptococcaceae*, and *Peptostreptococcales*. Both species diversity analysis and relative abundance analysis indicated differences in CKD patients after SSKE treatment. After SSKE treatment, the abundance of pathogenic bacteria decreased significantly, while the abundance of probiotics increased significantly. This suggests that SSKE can reach the colon and enhance its ability to regulate intestinal microbiota, thereby treating CKD.

## 4 Discussion

The prevalence of CKD is increasing yearly, characterized by pathological changes such as glomerular dilatation, enlarged glomerular mesangium, accumulation of mesangial matrix, basement membrane thickening, interstitial inflammation, and eventually tubular fibrosis (Chen et al., 2019). These changes can lead to a progressive decline in renal function and, over time, the development of chronic renal failure. Current treatments offer only partial protection against the progression of CKD.

In Asian countries, Chinese herbal medicine has been widely used to treat chronic diseases (Zhao et al., 2020). Ongoing research into Chinese herbal medicine has produced increasing evidence supporting its clinical efficacy. Our proposed formulation of SSKE aligns with the principles of Chinese medicine for treating CKD. Our results showed that SSKE could reduce enteric-derived endotoxin and proteinuria levels and mitigate glomerular histological alterations in CKD rats. Additionally, SSKE can improve intestinal damage caused by CKD by repairing intestinal tight junctions. Through *in vivo* and immunofluorescence experiments, we confirmed that the therapeutic effect of SSKE on CKD is mediated by the NF-κB signaling pathway.

To determine the appropriate dose for our study, we applied low, medium, and high doses of SSKE based on the human-mouse dose conversion formula. High doses of SSKE demonstrated the most significant anti-inflammatory and tissue structure improvement effects in CKD rats, providing preliminary evidence for the therapeutic effect of SSKE on CKD and its potential mechanism. Increasing evidence supports the critical role of the gut-kidney axis in CKD progression. We conducted a study simultaneously investigating the gut and kidney to verify that SSKE can improve CKD by regulating the microbiota-gut-kidney axis. Our previous study showed that SSKE could influence CKD progression by targeting the intestine and restoring its physical barrier. We first observed the cell ultrastructure and pathological conditions of the intestine and kidney, finding that both suffered from tissue structure and pathological damage in CKD rats. This provided a structural basis for treating CKD based on the gut-kidney axis. The molecular mechanism involving the NF-κB signaling pathway in treating CKD through the gut-kidney axis was further examined. Results from *in vivo* and immunofluorescence studies confirmed the regulation of the NF-κB signaling pathway in the intestine and kidney and demonstrated that SSKE improves CKD by inhibiting this pathway. Additionally, substantial evidence supports that gut microbiota-derived metabolites are therapeutic targets for reducing uremic toxicity, highlighting the therapeutic potential of the gut microbiota in CKD research.

This study has some limitations. Firstly, although we have shown that SSKE plays a therapeutic role in CKD rats by regulating the NF-κB signaling pathway, further *in vitro* cell studies are needed to explore its mechanism. Secondly, each compound of SSKE has not been validated for its respective mechanism, which presents a significant challenge. Thirdly, our studies were conducted in animal experiments and clinical trials, but larger controlled clinical trials are necessary to further ensure the efficacy and safety of SSKE in treating CKD.

## 5 Conclusion

In summary, this study highlights gut-derived endotoxin and inflammatory responses as key factors inducing renal tubular injury and renal fibroblast activation, emphasizing the activation of the NF-κB signaling pathway via the gut microbiota-gut-kidney axis in the progression of CKD. Our findings demonstrate that SSKE improves the physical structure and pathological morphology of the intestine and kidney, attenuates the inflammatory response, and reduces the secretion of TNF-α and IL-6 cytokines in CKD through the NF-κB pathway. Additionally, SSKE decreases the level of gut-derived endotoxin and regulates the composition and abundance of intestinal microorganisms, thereby improving the progression of CKD. These results suggest that SSKE is a therapeutic agent that slows CKD progression and provides valuable insights for CKD treatment.

## Data Availability

The original contributions presented in the study are publicly available. This data can be found here: Figshare, DOI: 10.6084/m9.figshare.28062827 (https://figshare.com/articles/dataset/dx_doi_org_10_6084_m9_figshare_28062827/28062827).
