# Supplementary material for: Shenshuaikang enema restores the intestinal barrier and microbiota-gut-kidney axis balance to alleviate chronic kidney disease via NF-κB pathway
Source: Front Pharmacol. 2025 Jan 21;15:1453668. doi: 10.3389/fphar.2024.1453668 (PMC11790348; doi:10.3389/fphar.2024.1453668)

**Supplementary information for**

Shenshuaikang enema restores the microbiota-gut-kidney axis balance to alleviate chronic kidney disease via NF-κB pathway

***Supplementary Material***

**Table 1.** Components in Shenshuaikang enema (SSKE).

| Plant scientific name | Herbal name | Application area | time of collection | Chinese  name | Weight  Ratio |
| --- | --- | --- | --- | --- | --- |
| *Astragalus membranaceus（Fisch.）Bge.* | *Astragali radlx* | Dried root | spring and  summer | Huangqi | 1 |
| *Rheum palmatum L.* | *Rhei radix et rhizoma* | Dried root and rhizome | late autumn | Dahuang | 1 |
| *Salvia miltiorrhiza Bge.* | *Salviae miltiorrhizae radix et rhizoma* | Dried root and rhizome | spring and  summer | Danshen | 1 |
| *Carthamus tinctorius L.* | *Carthami flos* | Dried flower | summer | Honghua | 1 |

**Table 2.** Mobile phase condition of chromatographic separation.

| Time | A% (Phosphoric acid) | B% (Methanol) |
| --- | --- | --- |
| 0 ~ 5 min | 5 | 95 |
| 5 ~ 10 min | 5 ~ 15 | 95 ~ 85 |
| 10 ~ 13 min | 15 | 85 |
| 12 ~ 15 min | 15 ~ 25 | 85 ~ 75 |
| 15 ~ 16 min | 25 ~ 45 | 75 ~ 55 |
| 16 ~ 23 min | 45 ~ 80 | 55 ~ 20 |
| 23 ~ 26 min | 80 ~ 100 | 20 ~ 0 |
| 26 ~ 30 min | 100 | 0 |

**FIGURE S1** The HPLC spectra of SSKE.


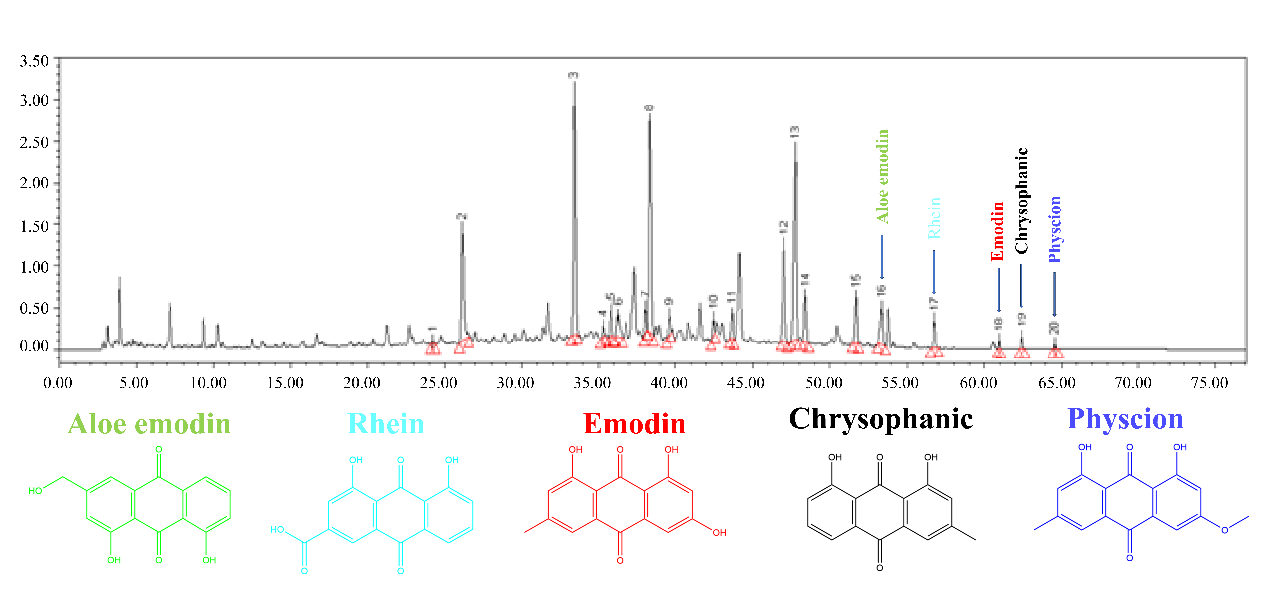


**FIGURE S2** The parameters of Scr, eGFR levels for all the patients (n=10) and rats (n=5). Data are represented as the mean ± SD (n=3). **p* < 0.05; ***p* < 0.01.


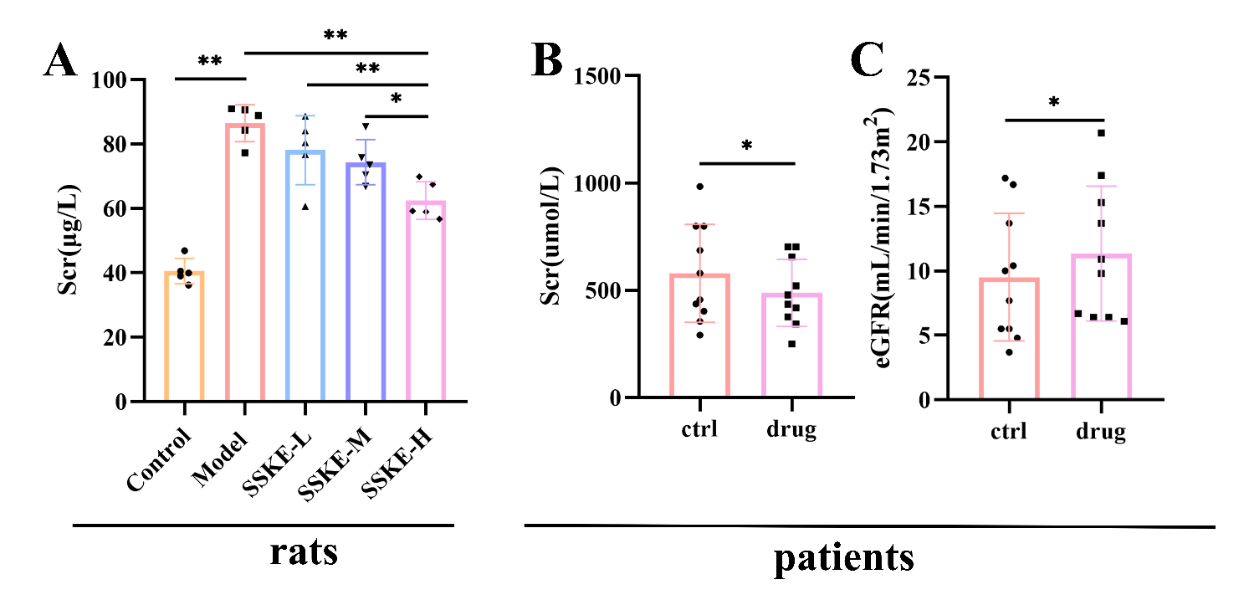

Supplement: Supplementary file 1 [file DataSheet1.docx]
